# Editorial: Eating disorders and eating disorder awareness

**DOI:** 10.3389/fnut.2025.1769959

**Published:** 2026-01-16

**Authors:** Mona Vintila, Cosmin Goian, Sebastian G. Soneira

**Affiliations:** 1 Department of Psychology, Faculty of Psychology and Education Sciences, West University of Timisoara, Timisoara, Romania; 2 Department of Social Work, Faculty of Sociology and Social Work, West University of Timisoara, Timisoara, Romania; 3 Department of Psychiatry, Fundacion Para la Lucha Contra las Enfermedades Neurologicas de la Infancia (FLENI), Buenos Aires, Argentina

**Keywords:** Anorexia and Bulimia, Binge Eating Disorder, diet, eating disorders, eating disorders prevention, food guidelines

Eating disorders, as highlighted by the Diagnostic and Statistical Manual of Mental Disorders, Fifth Edition (DSM-V), represent a significant and growing concern within the field of mental health. The four most prevalent eating disorders—Anorexia Nervosa, Bulimia Nervosa, Binge Eating Disorder (BED), and Avoidant/Restrictive Food Intake Disorder (ARFID)—are increasing, with some studies suggesting they have reached “epidemic” status.

This Research Topic has attracted a considerable number of interesting papers, covering diverse areas and utilizing a variety of approaches, including systematic reviews, exploratory analysis, psychosocial profiles, validation of psychometric instruments, interventions, and case studies. The research flow is comprehensive, addressing everything from health literacy and dietary knowledge to the role of socio-cultural factors, the relationship with body image, and subsequent implications for weight loss. The papers also examine interventions such as written emotional disclosure, bariatric surgery, or plastic surgery, as well as the evaluation of orthorexia, rounded out by interesting case studies.

## Key findings from reviewed studies

### Health literacy and sociodemographics

- The study “Eating disorders and health literacy in Germany” found that suspected eating disorders were more likely in female adolescents than male adolescents, but gender was not a determining factor in adults.
- The rate of suspected eating disorders increased with age in adolescents but decreased with age in adults.
- While education levels were not related to suspected eating disorders, low social status was associated with higher rates in adults, but not in adolescents.
- Inadequate or problematic health literacy and a negative body image were linked to higher rates of suspected eating disorders compared to adequate health literacy and a more positive body image.

## Dietary knowledge and psychosocial factors

- Research on Chinese college students showed a significant association between dietary knowledge based on the Chinese Dietary Guidelines and adherence to healthy dietary behaviors. This supports previous systematic reviews indicating that greater dietary knowledge tends to lead to healthier eating patterns in American and European populations.
- Among adolescents in Shandong Province, China, a higher psychosocial score was associated with a greater likelihood of maintaining a healthy dietary pattern.
- This association remained consistent across various demographic factors, including age, sex, residence, parental education, and family wealth. However, the ownership of a computer and access to the Internet modified the relationship between the psychosocial profile and the healthy dietary score.

## Body image and intentions

- The effect of body image on weight-loss intention among college students can be direct or indirect, mediated by self-efficacy and self-esteem (including a chained intermediary role).
- Body image and self-efficacy both have a significantly negative correlation with weight-loss intention. Conversely, self-esteem has a significantly positive correlation with weight-loss intention.
- A study on Chinese female university students found that body shape and BMI directly influence eating disorder behavioral intentions. The findings suggest that young Chinese women's eating disorder intentions are increasingly influenced by external factors related to body shape and BMI.
- Sociocultural standards promoting idealized appearance and the objectification of women's bodies are consistently linked to negative body image outcomes and an increased desire for cosmetic surgery. Research suggests that body image flexibility may act as a protective factor in reducing the desire for cosmetic surgery.

## Interventions and clinical complications

- Bariatric surgery is considered the most potent treatment for obesity and is believed to alleviate the symptoms of food addiction. A study of 78 patients found that bariatric surgery significantly and rapidly reduces food addiction scores, with improvements sustained for up to 2 years.
- While most food addiction symptoms remitted quickly, social/interpersonal problems, hazardous use, and large amount/longer use showed delayed improvement, suggesting distinct mechanisms for behavioral persistence.
- A systematic review showed that written emotional disclosure intervention is effective in alleviating eating disorder symptoms and improving patients' body image problems.
- Scurvy, a rare disease caused by vitamin C deficiency, can occur in individuals with restrictive eating disorders like Anorexia Nervosa (AN), leading to severe health complications. Patients with co-occurring AN and scurvy often present with gastrointestinal, psychiatric, and dermatological symptoms.
- The Chinese version of the Orthorexia Nervosa Inventory (ONI) was found to possess ^**^ strong reliability and validity^**^, making it a promising tool for assessing orthorexia tendencies and behaviors.

## Eating disorders in athletes

- The demanding physical requirements of football, which often involve maintaining a specific physique, can lead to harmful eating behaviors among professional female players due to internal and external pressures.
- Cultural norms influence the prevalence and types of eating disorders, with differences in eating habits, beauty standards, and socio-cultural pressures affecting their development.
- A study on Polish and Turkish professional female football players found no nationality-based differences in eating disorder prevalence, but found the disorder to be widespread, affecting about 40% of players.
- Nutritional status impacts ED risk, with higher risk found among both underweight and overweight players.
- A case study of an orienteering athlete with Anorexia Nervosa highlighted the critical importance of continuous monitoring, timely intervention, and a coordinated multidisciplinary team in addressing eating disorders in athletes.

## Conclusion

This Research Topic underscores the significance of effective communication among healthcare professionals and the necessity for comprehensive treatment strategies that include psychological, nutritional, and medical support in eating disorders. The studies collectively highlight the importance of early detection, suitable intervention, and the prevention of long-term health complications for effective eating disorder prevention.

